# Toward finding the difference between untreated celiac disease and COVID-19 infected patients in terms of CD4, CD25 (IL-2 Rα), FOXP3 and IL-6 expressions as genes affecting immune homeostasis

**DOI:** 10.1186/s12876-021-02056-1

**Published:** 2021-12-11

**Authors:** Nastaran Asri, Ehsan Nazemalhosseini Mojarad, Hamed Mirjalali, Seyed Reza Mohebbi, Kaveh Baghaei, Mohammad Rostami-Nejad, Abbas Yadegar, Mostafa Rezaei-Tavirani, Hamid Asadzadeh Aghdaei, Kamran Rostami, Andrea Masotti

**Affiliations:** 1 Basic and Molecular Epidemiology of Gastrointestinal Disorders Research Center, Research Institute for Gastroenterology and Liver Diseases, Shahid Beheshti University of Medical Sciences, Tehran, Iran; 2 Foodborne and Waterborne Diseases Research Center, Research Institute for Gastroenterology and Liver Diseases, Shahid Beheshti University of Medical Sciences, Tehran, Iran; 3 Gastroenterology and Liver Diseases Research Center, Research Institute for Gastroenterology and Liver Diseases, Shahid Beheshti University of Medical Sciences, Tehran, Iran; 4 Proteomics Research Center, Faculty of Paramedical Sciences, Shahid Beheshti University of Medical Sciences, Tehran, Iran; 5 Department of Gastroenterology, MidCentral DHB, Palmerston North, New Zealand; 6 Research Laboratories, Bambino Gesù Children’s Hospital-IRCCS, Rome, Italy

**Keywords:** SARS-CoV-2, COVID-19, Celiac disease, Gene expression, T-Lymphocytes, Regulatory, Interleukin-6

## Abstract

**Background:**

Coronavirus disease 2019 (COVID-19) is defined as an emerging infectious disease caused by severe acute respiratory syndrome coronavirus 2 and celiac disease (CD) is one of the autoimmune multiorgan diseases, which can be accompanied by an increased risk of viral infections. CD patients, especially untreated subjects, may be at greater risk of infections such as viral illnesses. Interleukin (IL)-6, CD4, CD25, and FOXP3 are known as genes affecting immune homeostasis and relate to the inflammation state. This study aimed to compare the expression levels of aforementioned genes in peripheral blood samples of CD and severe COVID-19 patients.

**Methods:**

Sixty newly diagnosed CD patients with median age (mean ± SD) of 35.40 ± 24.12 years; thirty confirmed severe COVID-19 patients with median age (mean ± SD) of 59.67 ± 17.22, and 60 healthy subjects with median age (mean ± SD) of 35.6 ± 13.02 years; were recruited from March to September 2020. Fresh whole blood samples were collected, total RNA was obtained and cDNA synthesis was carried out. RNA expression levels of IL-6, CD4, CD25, and FOXP3 genes were assessed using real-time quantitative RT-PCR according to the 2^−∆∆Ct^ formula. Statistical analysis was performed using SPSS (V.21) and GraphPad, Prism (V.6).

**Results:**

While increased expression of CD4, CD25, and FOXP3 was observed in CD patients compared to the control group (*p* = 0.02, *p* = 0.03, and *p* < 0.0001 respectively) and COVID-19 patients group (*p* < 0.0001 for all of them), their expression levels in COVID-19 patients decreased compared to controls (*p* < 0.0001, *p* = 0.01, *p* = 0.007, respectively). Increased IL-6 expression was observed in both groups of patients compared to controls (*p* < 0.0001 for both of them).

**Conclusions:**

Although untreated CD patients may be at greater risk of developing into severe COVID-19 if they are infected by SARS-CoV-2 virus (due to their high expression of IL-6), increased expression of anti-inflammatory markers in these patients may be beneficial for them with the ability of reducing the severity of COVID-19 disease, which needs to be proven in future studies involving celiac patients infected with COVID-19.

## Background

Celiac disease (CD) is a global, chronic, immune-mediated gluten-sensitive enteropathy elicited by dietary gluten in genetically predisposed individuals [1]. CD can induce various gastrointestinal (i.e., diarrhea, bloating, and abdominal pain) and extra-intestinal (i.e., iron deficiency anemia, osteoporosis, and weight loss) manifestations, or it may not be accompanied by any symptoms [2]. Gluten consumption changes the pro- and anti-inflammatory states and rises the production of pro-inflammatory cytokines leading to small intestinal inflammation in CD patients, which involves both innate and adaptive immune responses [3].

Interleukin 6 (IL-6) is a pleiotropic cytokine produced by different cell types and has both pro- and anti-inflammatory effects [4]. IL-6, as a pro-inflammatory agent, can stimulate the synthesis of acute-phase reactant proteins, and its overproduction is associated with inflammatory autoimmune disorders and uncontrolled intestinal inflammation (which takes place in CD) [5–7]. Numerous studies have linked IL-6 to the development of celiac disease [8].

Moreover, anti-inflammatory mediators, most importantly regulatory T cells (Tregs), can migrate into inflamed sites and attempt to minimize inflammatory responses [9]. The recruitment of Tregs is known as a key suppressive mechanism in maintaining peripheral immune homeostasis in chronic inflammatory diseases like CD [10].

Coronavirus disease 2019 (COVID-19) is defined as an emerging infectious disease caused by severe acute respiratory syndrome coronavirus 2 (SARS-CoV-2), which leads to a range of clinical manifestations including respiratory symptoms, fever, cough, kidney failure, or it can even be asymptomatic [11–13]. COVID-19 causes an ongoing global pandemic, as first identified in Wuhan, China, in December 2019 from a zoonotic source [11–13]. In general, the pathogenicity of a virus is dependent upon interactions between the pathogen and host pro- and anti-inflammatory mediators [14]. The interplay between innate and adaptive immune responses is involved in the development of COVID-19 disease. In fact, COVID-19 has an inflammatory pathophysiology involving a cytokine storm and it has been reported that during this infection the level of various analytes can change [15]. The unpredictability of the clinical course of the disease reinforces the need to find biomarkers that can help identify patients who are more vulnerable to clinical deterioration and improve patients’ management [16]. Previous studies investigated different biomarkers such as C-reactive protein (CRP), interleukin (IL)-6, procalcitonin (PCT), etc. in COVID-19 patients and their association with disease progression [15]. Among them IL-6 has attracted particular attention.

Several studies reported the significant role of IL-6 in the progression or suppression of viral infections [17]. Dysregulation of IL-6 was found to be related to the COVID-19 progression, and previous findings have reported its association with the severity of the disease, respiratory failure, and mortality in this group of patients [6, 18, 19]. In fact, the proper production of pro-inflammatory markers like IL-6 by various cell types after SARS-CoV-2 infection is positive in resolving the viral infection, that happens as a result of bacterial infections self-limitation due to human immune responses [20].

Tregs are important factors in controlling inflammation and preventing tissue complications during acute viral infections [19]. Anghelina et al. [21] reported that depletion of Tregs by treatment with anti-CD25 mAb (mAb PC61) from mice infected with murine coronavirus (rJ.M_Y135Q_-infected mice) resulted in a higher mortality rate with acute encephalitis. It is worth noting that, an important reason why obesity is reported as an unfavorable prognostic marker for COVID-19 is due to a decreased number of Tregs in the circulation and visceral adipose tissues in obese subjects [22]. Tregs express numerous receptors and non-receptor molecules on their surface that can be used as hallmarks to identify these cells. These markers include CD4 (one of the first markers expressed by Tregs), CD25 (essential for Tregs differentiation), and Forkhead box P3 (FOXP3) transcription factor (required for Tregs development and suppressive activity) [23–26].

It is noteworthy that CD patients may be at greater risk of infections such as viral illnesses [27, 28]. Although the results of studies indicate that there is not any increased risk of severe COVID-19 infection in gluten-free diet treated CD patients, little is known about untreated subjects, which caused concern among this group of patients and health care system [29, 30]. Due to the important role of IL-6 and Tregs in celiac disease and coronavirus infection, and owing to the importance of Treg markers in their development and activity, this study aimed to evaluate the gene expression of IL-6, CD4, CD25, and FOXP3 in peripheral blood samples of newly diagnosed CD patients and COVID-19 infected people, relative to healthy subjects, to compare their inflammation state. In fact, we conducted this study to compare the inflammatory status of active celiac patients with severe COVID-19 infected subjects to evaluate if newly diagnosed or even undiagnosed CD patients are at increased risk of severe COVID-19 infection.

## Methods

### Clinical samples

60 newly diagnosed CD patients who were not infected with the COVID-19, median age (mean ± SD) 35.40 ± 24.12 years; 23 males and 37 females, and 30 confirmed severe COVID-19 patients, median age (mean ± SD) 59.67 ± 17.22 years; 20 males and 10 females, considered as patient groups. Patients were admitted to Taleghani hospital (Tehran, Iran) from March to September 2020. In addition, sixty healthy subjects, median age (mean ± SD) 35.6 ± 13.02 years; 27 males and 33 females, with no history of CD and other immune-related diseases who were not infected with COVID-19 and had no contact with infected people were used as controls.

The confirmed cases of COVID-19 (based on clinical manifestations, radiology/CT scan, molecular detection by qPCR), who were within a week of developing symptoms, and confirmed untreated CD patients (according to serological and histopathological (Marsh classification) criteria) [27], were included in this study. The following patients were excluded from the study: CD patients who had another autoimmune disorder, COVID-19 patients with any autoimmune disorder, and patients who used any prohibited medications. Healthy individuals who had not current or prior history of gastrointestinal disorders and COVID-19 infection, and females who were not pregnant were included. Fresh whole blood (10 mL) samples were collected from patients belonging to the above-described studied groups in EDTA anticoagulant tubes using standardized venipuncture.

### Ethics statement

We obtained approval from the Ethics Committee of Shahid Beheshti University of Medical Sciences, Tehran, Iran (IR.SBMU.RETECH.REC.1399.088). Written informed consent was obtained from patients before they participated in the research.

### RNA extraction and cDNA synthesis

Total RNA was obtained from peripheral blood mononuclear cells (PBMCs) using the Total RNA Purification Mini kit for Blood/Cultured Cell/Tissue (Yekta Tajhiz Azma, Tehran, Iran) according to the provided protocol. Isolated RNA quantity and purity were determined by spectrometry (Nanodrop, Thermo Scientific NanoDrop Products, Wilmington, DE, USA) and gel electrophoresis. Seven micrograms of total RNA were used for cDNA synthesis using the 2 Step 2X RT-PCR Premix (Taq) kit (BioFact™, South Korea), according to the manufacturer instructions in total 20 μL reaction mixture. RT reaction conditions were used: 25 °C for 5 min, 50 °C for 30 min, and 95 °C for 5 min.

### Gene expression analysis

Appropriate primers for IL-6, CD4, CD25, FOXP3, and Glyceraldehyde 3-phosphate dehydrogenase (GAPDH), as internal control, were designed and analyzed by Gene Runner v. 3.05 software (Table 1). In the following, the specificity of the primers was checked by performing PCR experiments. The reaction was immediately started by a denaturation step for 5 min at 95 °C, followed by 40 cycles of 40 s at 94 °C, 30 s at 57–62 °C (Temperature gradient), and 30 s at 72 °C, and a final extension step for 10 min at 72 °C. Products were separated by electrophoresis on a 1.5% agarose gel, and bands were visualized using UV-fluorescence.

**Table 1:** Specific primers used for real-time quantitative PCR

Gene symbol	Primer sequence	Length	Product length (bp)
*CD4*	F:5′-ACATCAAGGTTCTGCCCAC-3′	19	190
	R:5′-TGGCAGGTCTTCTTCTCAC-3′	19	
*CD25*	F:5′-ACTTCCTGCCTCGTCACAAC-3′	20	174
	R:5′-ACTCTTCCTCTGTCTCCGCT-3	20	
*FOXP3*	F:5′-TCATCTGTGGCATCATCCG-3′	19	167
	R:5′-AGGAACTCTGGGAATGTGC-3′	19	
*IL-6*	F:5′-GATTCAATGAGGAGACTTGCC-3′	22	132
	R:5′-GGTCAGGGGTGGTTATTGC-3′	22	
*GAPDH*	F:5′-TGTGGGCATCAATGGATTTGG-3′	21	116
	R:5′-ACACCATGTATTCCGGGTCAAT-3′	22	

The messenger RNA (mRNA) expression of the target genes was determined using real-time quantitative RT-PCR according to the 2^−∆∆Ct^ formula (ΔCt = Ct^gene^–Ct^control^). Each sample was run in duplicates on a Rotor-Gene Q real-time PCR system (QIAGEN) using 10 μL of 2X Real-Time PCR Smart mix Sybergreen (BioFact™, South Korea), 1 μL cDNA, 0.5 μL of each forward and reverse primers, and 8 μL of H2O in a final volume of 20 μL. The cycling program started with heating at 95 °C for 15 min followed by 40 cycles of a three-stage temperature profile of 95 °C for 10 s, 56 °C (CD4 and IL-6) and 60 °C (CD25 and FOXP3) for 40 s, and 72 °C for 30 s. The workflow was controlled for contamination completely.

### Statistical analysis

Statistical analysis was performed using SPSS (V.21) and GraphPad, Prism (V.6). The significance of differences in averaged gene expressions was analyzed using One-way ANOVA followed by the Tukey test. *p* values ≤ 0.05 were considered significant.

## Results

### Clinical characteristics of study subjects

**Fig. 1:**
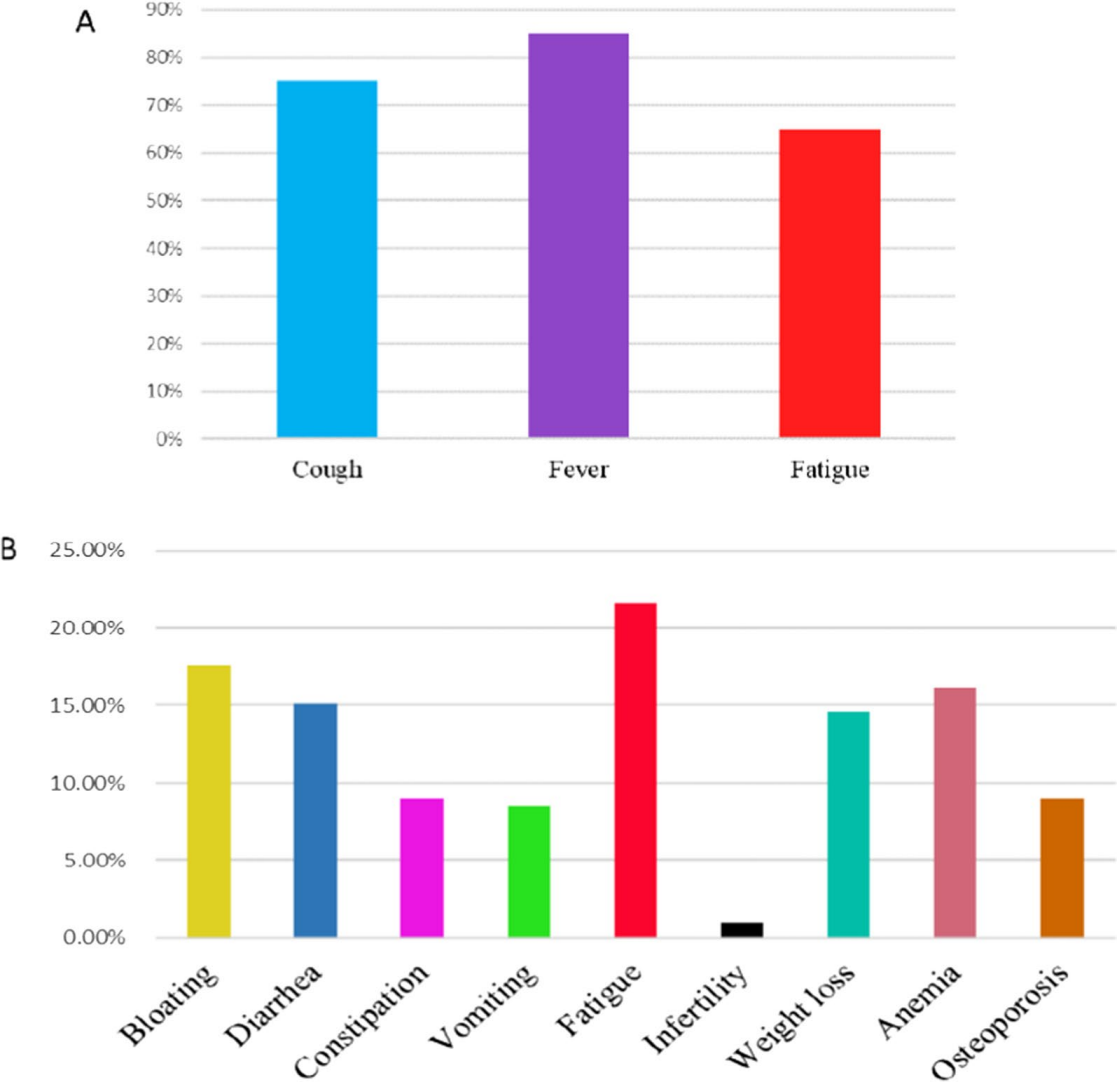
Symptoms reported by COVID-19 and CD patients: most patients complained of two or more symptoms. **A** COVID-19 patients, **B** CD patients symptoms. *COVID-19* Coronavirus disease 2019, *CD* celiac disease

Most of the COVID-19 patients presented with fever (26/30, 86.6%), dry cough (23/30, 76.6%), and fatigue (20/30, 66.6%). The most common gastrointestinal symptoms of CD patients were bloating (10/60, 16.6%) and diarrhea (9/60, 15%), and the most common extra-gastrointestinal symptoms were fatigue (13/60, 21.6%) and anemia (10/60, 16.6%) (Fig. 1).

### Gene expression analysis

**Fig. 2:**
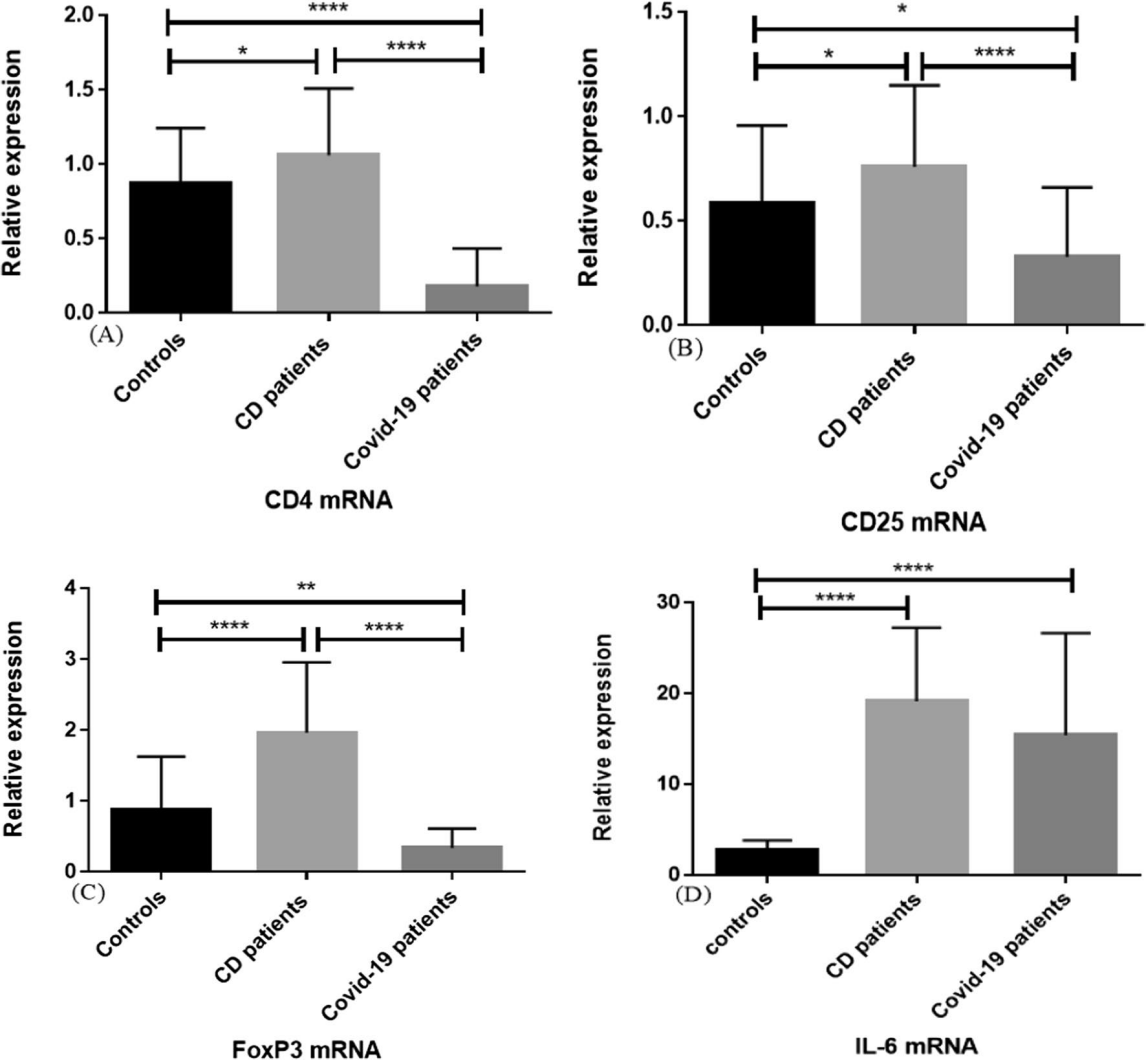
Analysis of relative expression levels in CD and COVID-19 patients compared to normal controls using real-time PCR assay. All expression levels are normalized to that of GAPDH. The analysed genes were as follows: **A** CD4; *p = 0.02, ****p < 0.0001, **B** CD25; *p = 0.01 (between COVID-19 patients and controls) and *p* = 0.03 (between CD patients and controls), ****p < 0.0001; **C** FOXP3; **p = 0.007, ****p < 0.0001; **D** IL-6; ****p < 0.0001. Data are presented as mean ± S.D. *CD* celiac disease, *COVID-19* coronavirus disease 2019, *GAPDH* glyceraldehyde 3-phosphate dehydrogenase, *FOXP3* forkhead box P3

The comparative threshold cycle (Ct) method was used to determine relative transcript levels of CD4, CD25, FOXP3, and IL-6 genes using GAPDH as a housekeeping control. The results showed that the relative expression levels of CD4, CD25, and FOXP3 mRNA were significantly increased in CD patients (mean ± SD: 1.05 ± 0.45, 0.75 ± 0.39, 1.96 ± 0.99, respectively) compared to the control group (mean ± SD: 0.86 ± 0.37, 0.58 ± 0.37, 0.87 ± 0.75, respectively), (*p* = 0.02, *p* = 0.03, and *p* < 0.0001 respectively) and COVID-19 patients’ group (mean ± SD: 0.18 ± 0.25, 0.32 ± 0.33, 0.33 ± 0.27, respectively), (*p* < 0.0001 for all of them). On the contrary, the relative level of CD4, CD25 and FOXP3 mRNA expression in COVID-19 patients was significantly lower than in controls (*p* < 0.0001, *p* = 0.01, *p* = 0.007, respectively) (Fig. 2). Moreover, the expression level of IL-6 exhibited a significant increase in both CD (mean ± SD: 19.16 ± 8.11) and COVID-19 patients (mean ± SD: 15.42 ± 11.23) in comparison to controls (mean ± SD: 2.81 ± 1.02), (*p* < 0.0001 for both of them), but there was no significant difference in IL-6 level between these two groups of patients (Fig. 2).

## Discussion

COVID-19 is a viral disease that may trigger an inflammation similar to CD and is associated with impaired immune tolerance [31–33]. Celiac disease is one of the autoimmune multiorgan diseases, which can be accompanied by an increased risk of viral infections [27]. While preliminary results suggest that GFD-treated CD patients are less likely to develop severe COVID-19 infection, it has been reported that their defective nutritional status (Folate, vitamin B_12_ and vitamin D), specially in those who are not treated with GFD, is associated with a higher risk of respiratory infections [29, 34]. Likewise, limited data are available on the risk of severe COVID-19 infection in untreated celiac disease patients [29].

IL-6, as a pro-inflammatory cytokine, is involved in inflammation and can adversely affect immune responses against virus infection [35–37]. Present data showed that the expression level of IL-6 is significantly increased in the PBMC of both severe COVID-19 and CD patients in comparison to controls (*p* < 0.0001 for both of them). In this regard, in a meta-analysis study conducted on a total of 1426 COVID-19 patients it has been demonstrated that, mean IL-6 levels were extremely higher in complicated COVID-19 patients in comparison to those with non-complicated disease [38]. Studies considered this cytokine as a potential predictor of COVID-19 severity, respiratory failure and mortality risk [19, 39, 40]. In celiac disease, IL-6 stimulates acute phase responses and promotes T-helper cell 17 (Th17) differentiation, which leads to tissue injury [41]. Studies showed an increase in serum levels of IL-6 in untreated CD patients following gluten intake and its decrease after one-year gluten elimination from the diet [5]. Accordingly, Romero-Adrián et al. demonstrated an appropriate correlation between IL-6 and CD activity and found it as a suitable marker for detecting minimal contamination with gluten-containing substances [42]. Noting that the patients in this study were also in the active phase of the disease, the increase in IL-6 levels is reasonable. In fact, the overproduction of pro-inflammatory cytokines like IL-6 is associated with autoimmune disorder, and lead to cytokine storm, what is reported in severe COVID-19 patients [43].

The expression levels of CD4, CD25 and FOXP3 showed a significant decrease in severe COVID-19 patients’ PBMC compared to controls (*p* < 0.0001, *p* = 0.01, *p* = 0.007, respectively) and CD patients (*p* < 0.0001 for all of them). Conversely, a significant increase in expression levels of these markers was shown in the CD patients’ PBMC specimens related to healthy controls (*p* = 0.02, *p* = 0.03, and *p* < 0.0001 respectively). These results complement data from previous studies [22]. Han and co-workers [44] showed increased peripheral blood CD4+ T cell numbers in CD patients who were on a gluten-free diet in response to oral gluten challenge. Vorobjova et al. [45] also reported that the majority of CD patients' intestinal lymphocytes were CD4 cells. Considering that gluten-specific CD4+ T cells also play a role in driving the pathogenic immune responses in CD, CD4 increased expression may indicate both disease progression and suppression [10, 46]. Tiittanen et al. [47] observed an increased number of CD25+ cells in the intestinal specimens of children with active CD. Penttila and colleagues [48] also showed increased expression of IL-2R on PBMCs from CD patients in response to stimulation with gluten fraction. These results are in line with the finding of our study. Moreover, Frisullo et al. [49] reported increased expression of FOXP3 in peripheral blood CD4+ CD25+ T cells of untreated CD patients compared to treated patients. As FOXP3 expressing Tregs have an important role in maintaining the intestinal hemostasis balance, the increased FOXP3 gene expression by our CD patients makes sense [50, 51].

Current reports on the number of Treg cells and their markers expression in COVID-19 patients remains controversial. Although plenty of studies indicate the increased Treg cells and markers in patients with COVID-19 (especially those with the milder disease), their significant reduction in PBMCs of severe COVID-19 patients was also reported in other studies [52]. Ong et al. [53] and Diao and colleagues [54] linked the decreased blood expression of CD4 in COVID-19 patients to severe form of the disease. Mohebbi et al. [55] demonstrated a downregulated PBMC expression level of CD4 and CD25 in COVID-19 patients in comparison to healthy controls that was in line with our results. It is important to note that CD25 deficiency is reported to be accompanied by increased susceptibility for chronic viral infections [56]. Decreased levels of FOXP3 mRNA and its association with increased IL-6 transcription level in COVID-19 infection, which was observed in our study, have also been reported by Mohebbi et al. [53]. In a recent study, decreased number of Tregs with a decreased expression of FoxP3 mRNA and immunosuppressive cytokines (IL-10 and TGFβ) was reported in PBMC derived from the COVID-19 patients treated in ICU [54]. Authors declared that, these reductions in severe cases of the COVID-19 may contribute to the excessive production of pro-inflammatory cytokines that leads to ARDS [50].

In case of CD, as the pro-inflammatory markers like IL-6 are already present, the pre-existing autoimmune reaction may help to curb the proliferation of SARS-CoV-2 virus when the CD patients are contacted with the SARS-CoV-2 virus, makes the COVID-19 cases of CD patients to be mild if the CD patients do not have metabolic disorders like obesity or diabetes mellitus. Yet, the present of SARS-CoV-2 viral infection may further worsen the autoimmune disorder in CD patients [43]. In other words, it can be said that the high level of anti-inflammatory mediator markers observed in untreated patients with celiac disease, may prevent them from developing into severe COVID-19 if they are infected by SARS-CoV-2 virus. Further studies are needed to confirm or refute our hypothesis.

## Conclusions

The results of this study can provide clinically useful information about CD and COVID-19 infection patients’ inflammation state. In our opinion, although high levels of IL-6 can be detrimental to untreated CD patients and predispose them to severe COVID-19 infection if they are infected by SARS-CoV-2 virus, increased expression of CD4, CD25 and FOXP3 as anti-inflammatory markers in these patients may be beneficial for them with the ability of reducing the severity of COVID-19 disease, which needs to be proven in future studies involving celiac patients infected with COVID-19. The limitation of our study is not to include CD patients which tested positive for COVID-19 and therefore, further studies are needed to confirm/ reject the role of anti-inflammatory markers in protecting celiac patients from COVID-19 infection.

## Data Availability

All data generated or analyzed during this study are included in this published article. The datasets used and analyzed during the current study are not publicly available due to our center’s patient confdentiality policies, but they are stored in the Gastroenterology and Liver Diseases Research Center, Research Institute for Gastroenterology and Liver Diseases, Shahid Beheshti University of Medical Sciences repository and are available from the corresponding author on reasonable request.

## Abbreviations

CD: Celiac disease
COVID-19: Coronavirus disease 2019
CT: Comparative threshold
FOXP3: Forkhead box P3
GAPDH: Glyceraldehyde 3-phosphate dehydrogenase
IL: Interleukin
mRNA: Messenger RNA
PBMCs: Peripheral blood mononuclear cells
SARS-CoV-2: Severe acute respiratory syndrome coronavirus 2
Th17: T-helper cell 17
Tregs: Regulatory T cells
